# Second-generation non-hematopoietic erythropoietin-derived peptide for neuroprotection

**DOI:** 10.1016/j.redox.2021.102223

**Published:** 2021-12-21

**Authors:** Bongki Cho, Seung-Jun Yoo, So Yeon Kim, Chang-Hun Lee, Yun-Il Lee, Seong-Ryong Lee, Cheil Moon

**Affiliations:** a Department of Brain & Cognitive Sciences, Graduate School, DGIST, Daegu, 42988, South Korea; b Convergence Research Advanced Centre for Olfaction, DGIST, Daegu, 42988, South Korea; c Division of Biotechnology, DGIST, Daegu, 42988, South Korea; d Department of New Biology, DGIST, Daegu, 42988, South Korea; e New Biology Research Center, DGIST, Daegu, 42988, South Korea; f Department of Pharmacology and ODR Center, Brain Research Institute, School of Medicine, Keimyung University, Daegu, 42601, South Korea

**Keywords:** Erythropoietin, Erythropoietin receptor, Peptide, Neuroprotection, Hypoxia, Ischemia, Erythropoietin, EPO, erythropoietin receptors, EPORs, recombinant EPO, reEPO, Janus kinase 2, JAK2, signal transducer and activator of transcription 5, STAT5, ERK, Extracellular signal-regulated kinases 1/2, fetal bovine serum, FBS, nerve growth factor, NGF, genetic algorithm 341, G341, 2′,7′-dichlorodihydrofluorescein diacetate, H_2_DCFDA, 2,3,5-triphenyltetrazolium chloride, TTC

## Abstract

Erythropoietin (EPO) is a well-known erythropoietic cytokine having a tissue-protective effect in various tissues against hypoxic stress, including the brain. Thus, its recombinants may function as neuroprotective compounds. However, despite considerable neuroprotective effects, the EPO-based therapeutic approach has side effects, including hyper-erythropoietic and tumorigenic effects. Therefore, some modified forms and derivatives of EPO have been proposed to minimize the side effects. In this study, we generated divergently modified new peptide analogs derived from helix C of EPO, with several amino acid replacements that interact with erythropoietin receptors (EPORs). This modification resulted in unique binding potency to EPOR. Unlike recombinant EPO, among the peptides, ML1-h3 exhibited a potent neuroprotective effect against oxidative stress without additional induction of cell-proliferation, owing to a differential activating mode of EPOR signaling. Furthermore, it inhibited neuronal death and brain injury under hypoxic stress *in vitro* and in an *in vivo* ischemic brain injury model. Therefore, the divergent modification of EPO-derivatives for affinity to EPOR could provide a basis for a more advanced and optimal neuroprotective strategy.

## Highlights

• Short peptides derived from helix C of EPO have a neuroprotective effect.
• Divergent modification of EPO-derived peptides has a differential affinity to EPOR.
• ML1 and its analogs have differential cell protective and proliferative effects.
• ML1-h3 protects neurons by suppressing *in vitro* oxidative stress.
• ML1-h3 mitigates brain injury in the *in vivo* mouse ischemic model without hematopoietic effect.

## Graphical abstract

**Figure:**
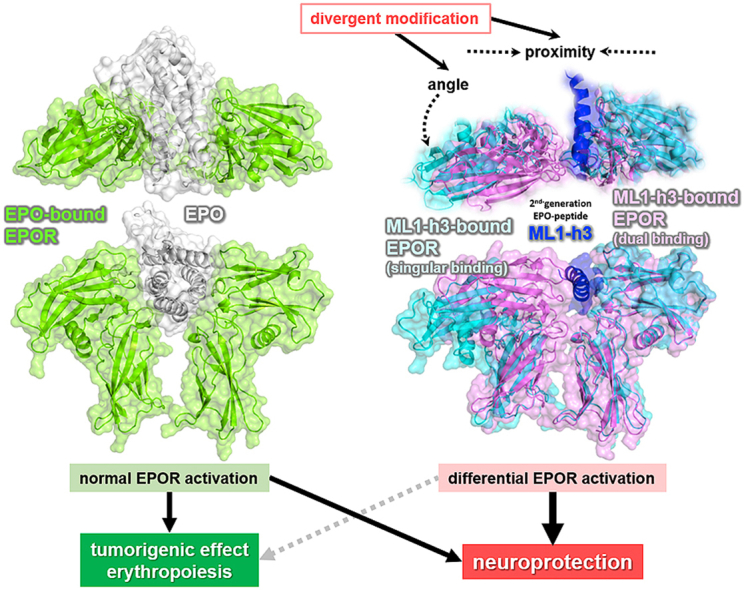

## 1 Introduction

Erythropoietin (EPO), a well-known erythropoietic cytokine, promotes survival, proliferation, and differentiation of erythroid progenitor cells to generate red blood cells [1]. Thus, direct administration of recombinant EPO is used to treat anemia [1]. In other organs, including the brain, kidney, heart, and muscle, it plays a tissue-protective role against hypoxic stress [2]. EPO has also been suggested as a potential neuroprotective compound in treating neurological disorders including stroke, multiple sclerosis, schizophrenia, retinopathy, epilepsy, Parkinson's disease, and Alzheimer's disease [[3], [4], [5]]. Although studies have demonstrated the neuroprotective effects in the *in vitro* and *in vivo* neurodegeneration models, the effects have been limited by their off-target erythropoietic and tumorigenic effects [6,7]. Therefore, abolishment or reduction of these off-target effects is required for EPO-based therapeutics.

Previously, to overcome these adverse effects, some neuroprotective variants of EPO have been developed [8]. Based on the sialylation-mediated inhibition of clearance of circulating EPO in the liver [9], rapidly cleared EPO-variants *in vivo*, such as asialoEPO and Neuro-EPO with decreased or absent sialic acids, have been suggested [10,11]. Recently, EPO-variants having modification or *de novo* addition on N-linked glycosylation have also been suggested [12,13]. They exhibit an efficient neuroprotective effect without additional erythropoiesis. Carbamylated EPO does not directly bind to EPO receptor (EPOR) but activates β common receptor (CD131); this forms a tissue-protective receptor complex with a single EPOR molecule, resulting in a tissue-protective effect without erythropoiesis [14]. These reports suggest that sustainability, intensity, and mode of EPOR activation may underlie EPO-induced adverse effects. According to structural biological studies, EPO consists of four α-helices (helix A, B, C, and D), which cluster into a relatively rigid globular structure by hydrophobic interaction [15]. One EPO molecule binds to two asymmetrical interfaces, including the high- and the low-affinity interfaces in the extracellular domain of dimeric EPOR_1_ and EPOR_2_, respectively [[16], [17], [18], [19]]. While the high-affinity interaction with EPOR_1_ occurs in helix D and the loop between helices A and B of EPO, the low-affinity interaction with EPOR_2_ occurs in helices A and C. Based on this structural property, for partial interactive interface in EPOR, EPO-derived short peptides, such as epopeptide AB, EPObis, JM-4, EPOtris, and NL100, have been suggested as neuroprotective agonists [[20], [21], [22], [23], [24]]. The short peptides derived from the helix B of EPO also induce tissue-protective effects without erythropoietic effect via the activation of CD131 similarly to carbamylated EPO [[25], [26], [27], [28]]. Notably, these short peptides do not show an erythropoietic effect. Recently, we also generated a novel peptide derived from helix C of EPO, MK-X, which only has neuroprotective effects without any cell-proliferative effects [29,30]. Taken together, partial binding to EPOR is not sufficient for EPOR-mediated erythropoiesis and cell proliferation. Therefore, tuning the binding mode or affinity of EPO-derivatives to EPOR can provide an important clue for an EPO-based neuroprotective strategy without the undesired erythropoietic and tumorigenic effects.

When EPO binds to dimeric EPOR, EPOR-bound Janus kinase 2 (JAK2) is auto-phosphorylated; this process leads to further activation of STAT5 (signal transducer and activator of transcription 5) signaling, noncanonical activation of AKT (protein kinase B), and ERK (Extracellular signal-regulated kinases 1/2) signaling cascades [[31], [32], [33]]. Given that these signaling pathways are critical for EPOR-mediated cell survival and proliferation [7], EPO-derivatives having only neuroprotective effects without adverse effects may differentially activate EPOR downstream signaling, unlike the recombinant EPO. We found that MK-X, a short peptide derived from helix C of EPO, exhibits a different activation mode for EPOR downstream signaling under oxidative stress [29,30]. However, the underlying mechanisms of only cell-protective effects of EPO-derivatives, unlike the recombinant EPO, remain unclear.

In this study, we generated divergently modified new peptides derived from helix C of EPO with differential affinity to EPOR. Thereby, this induced a unique mode activation of EPOR signaling. Especially, ML1-h3, a modified peptide, exhibited improved neuroprotective effects in the *in vitro* and *in vivo* conditions, without cell-proliferative and erythropoietic activities. Using this approach, we provide a basis for a novel EPO-based therapeutic strategy for neurodegeneration.

## 2 Material and methods

### 2.1 Peptides, recombinant EPO, and chemicals

All peptides including ML1, ML1-c1, ML1-c2, ML1-c3, ML1-h1, ML1-h2, and ML1-h3 were synthesized as monomers by solid-phase peptide synthesis. A negative control peptide (AMELSGRTLGLILKLRQSATL), which has scrambled sequences derived from helix C region of EPO, did not bind to EPOR, and unaffected cell survival and proliferation, was used as previously reported [30]. To evaluate the purity and concentration of the peptides, LC/MSD (HP 1100 series) and HPLC analyses (SHIMADZU prominence HPLC) were performed. We obtained peptides having >95% purity and then dissolved with DMSO or water as a highly concentrated stock solution (1 μg/μl or 10 mM). The peptide stock solutions were preserved at −80°C. Recombinant human EPO (reEPO; Epoetin-α, CJ HealthCare Corporation, South Korea) concentrated to 10000 IU/ml (2.74 μM) was provided by Prof. Seong-Ryong Lee in Keimyung University. It was preserved at −70°C till usage. All peptides were purchased from Peptron Inc. (Daejeon, Korea). We used a commercially purchased 30% H_2_O_2_ solution (#107209, Merck Millipore, Germany) and freshly prepared it for experiments by diluting 300 mM stock solution into water.

### 2.2 Cell culture

PC12 cells (#CRL-1721, ATCC, USA), a rat pheochromocytoma cell line derived from the adrenal medulla (#CRL-1721, ATCC, USA), were seeded on a dish, which was coated by poly l-lysine (0.01%; Sigma Chemical Co., USA), and grown in RPMI 1640 medium (#11875093, Gibco, USA), supplemented with 10% heat-inactivated horse serum (#26050088, Gibco, USA), 5% fetal bovine serum (FBS) (#SH30919.03, Hyclone, USA), and 1% penicillin/streptomycin at 37°C with 5% CO_2_. For neuronal differentiation, PC12 cells were plated at a density of 1.25x10^3^ cell/cm^2^ into 96-well plates coated with collagen 1 (#354236, BD Biosciences, USA) and grown in RPMI 1640 medium supplemented with 50 ng/ml nerve growth factor (NGF) (BD Biosciences, USA) and 0.5% FBS for 10 days. The NGF-supplemented media was replaced with fresh media every 3 days during neuronal differentiation.

HT-22 cells (kindly provided by Prof. Gil-Saeng Jeong in Keimyung University), an immortalized mouse hippocampal cell line, were cultured at 37°C and 5% CO_2_ in Dulbecco's modified eagle medium (DMEM, Invitrogen, USA) supplemented with 10% FBS and 1% penicillin/streptomycin at 37°C and 5% CO_2_.

### 2.3 Rat primary cortical neuron culture

On the embryonic day 17, embryonic cerebral cortices were dissected from pregnant Sprague–Dawley rats. The cerebral cortices were treated with 0.25% trypsin-EDTA (#25200056, Gibco, USA) and then mechanically dissociated into single cells by pipetting. The dissociated cells were plated on 96-well plates coated by 50 μg/ml poly-l-lysine (P8920, Sigma-Aldrich, USA) at a density of 2.5 × 10^4^ cells per well. They were incubated in neurobasal medium (#21103049, Gibco, USA) containing 2% B27 supplement (#17504044, Gibco, USA), 0.5 mM l-glutamine (#15430614, Gibco, USA), 25 μM glutamic acid (#G1251, Sigma-Aldrich, USA), and 1% penicillin/streptomycin (#15140122, Gibco, USA) at 37°C with 5% CO_2_. The medium was changed to a fresh medium without glutamate every 3–4 days*,* and on day 10, glutamic acid (30 μM) was added to induce excitotoxicity in the cells treated with or without reEPO and peptides.

### 2.4 Molecular modeling and analysis

All models were built using MODELLER [34] with the crystal structure of EPO and EPOR complex [35] (PDB: 1EER) as a template. Modeling studies showed binding between peptides and EPOR where 100 models were generated for each complex structure with a corresponding peptide ligand, producing both singular binding model with only EPOR_2_ and dual binding model with EPOR_1_ and EPOR_2_, simultaneously, in the same modeling process. The best models were selected based on the structural quality parameters of discrete optimized protein energy (DOPE) and genetic algorithm 341 (G341) scores [36,37]. Visualization, manipulation, and analysis of the molecular model were performed using the PyMOL program (The PyMOL Molecular Graphics System, Version 2.0 Schrödinger, LLC.). To visualize the electrostatic potential on the molecular surface of peptides, we used the ‘generate-vacuum electrostatics-protein contact potential’ module of the Pymol program. To examine the geometrical property of EPOR dimers, we aligned the structures of EPO- and peptide-bound EPORs based on peptide and EPOR_2_ and performed structural measurements by using the ‘measurement’ module in the PyMOL program. As previously described [38], proximity between the dimeric EPORs was assessed by measuring the distance between Cαs values of ^143^Val in EPOR_1_ and EPOR_2_, the structural hinges of the extracellular domain. To measure a change in the angle between EPORs by peptide binding as compared to that between EPOs, we extended the first line between Cαs of ^143^Val in EPO-bound EPOR_1_ and EPOR_2_, and the second line between Cαs of ^143^Val in EPO-bound EPOR_2_ and peptide-bound EPOR_1_; the angles between the two lines were measured. In addition, potential polar and proximal contacts within 4 Å were visualized using the ‘find’ module in the PyMOL program.

### 2.5 Surface plasmon resonance analysis

Real-time surface plasmon resonance analysis was performed using the Reichert SPR Biosensor SR 7500 C instrument (Reichert Inc., NY, USA). Soluble recombinant human EPOR-Fc chimera protein (#963-ER, R&D Systems, Minneapolis, MN, USA) was covalently immobilized on a carboxymethylated dextran matrix-coated chip (BR-1005-39, Pharmacia Biosensor AB) by using an amine coupling kit (BR-1000-50, GE healthcare) according to the manufacturer's instructions. Each concentration (5, 2.5, and 1.25 μM) of the peptide flowed at 5 μl/min was tested in duplicate. After each binding cycle, the sensor chip was recharged by 20 μl/min injections of 25 mM acetic acid.

### 2.6 Measurement of cell viability, proliferation, and ROS levels

Cell viability was evaluated by 3-[(4,5-dimethylthiazol-2-yl)-5,3-carboxymethoxyphenyl]-2-(4-sulfophenyl)-2H tetrazolium, inner salt (MTS) reduction assay (CellTiter 96 Aqueous One Solution Cell Proliferation Assay, Promega, Madison, WI, USA). Briefly, differentiated neuronal-PC12 cells were treated with H_2_O_2_ (300 μM) with or without reEPO and peptides for 24 h. The PC12 cells were washed with PBS; 20 ul MTS solution was added to each well with 100 μl PBS, and the cells were incubated for 3 h at 37°C and 5% CO_2_. The intracellular soluble formazan produced by cellular reduction of the MTS was determined by recording the absorbance at 490 nm using a microplate reader (VersaMax, Molecular Devices, USA). Cell proliferation was also evaluated by the MTS assay. Undifferentiated PC12 cells were seeded into a 96-well plate (5x10^4^ cells/well). The next day, the initial cell number was counted; after 48 h, under low FBS concentration (0.5%), with or without reEPO and peptides, cell number was measured by the MTS assay.

The cellular ROS level was measured using 2′,7′-dichlorodihydrofluorescein diacetate (H_2_DCFDA) (#D399, Invitrogen, USA). The cells were incubated with 5 μM H_2_DCFDA in PBS for 30 min, washed with PBS, and then resuspended in the medium for 2 h. After treatment of H_2_O_2_ without or with reEPO, or peptide, we monitored the oxidative stress by measuring the fluorescence intensities of H_2_DCFDA by using excitation and emission wavelengths of 485 nm and 535 nm, respectively.

To measure the viability of rat primary cortical neurons, we used Calcein-AM (#C3100MP, Invitrogen, USA). Cultured rat cortical neurons plated on 96-well plates (2.5 × 10^4^ cells per well) were incubated with Calcein-AM (3 μM) for 30 min after various treatments. The intensity of the Calcein-AM signal (excitation, 485 nm; detection 535 nm) was measured by using a micro plate reader (VersaMax, Molecular Devices, USA).

### 2.7 Circular dichroism spectroscopy

ML1-h3 peptide (100 μM) is resolved in distilled water containing 0%, 10%, 20%, 40%, 60% or 80% 2,2,2-Trifluoroethanol (#T63002, Sigma-Aldrich, USA). Each sample was loaded into a 0.1 mm cuvette, and circular dichroism (CD) spectra were recorded using a circular dichroism spectrophotometer (#J-1500, JASCO, USA) at 20°C. CD spectra were recorded in 0.1 nm intervals with a scanning speed of 20 nm/min, and the photomultiplier voltage was from 300 V at 190 nm to 200 V at 250 nm. CD spectra from each sample were taken in triplicate, averaged, and then subtracted from the baseline CD spectrum of each vehicle solution without peptide. Normalization of CD spectra data and prediction of the secondary structure of ML1-h3 was performed by the BeStSel method (https://bestsel.elte.hu/index.php) [[39], [40], [41]].

### 2.8 Immunoblotting and antibodies

PC12 or HT-22 cells were lysed in 100 mM Tris-Cl (pH 6.8) with 4% SDS. The lysates were loaded onto sodium dodecyl sulfate-polyacrylamide gel electrophoresis (SDS-PAGE) (8–10%), and the proteins were transferred onto the nitrocellulose membrane. The membrane was blocked with 0.1% TBST and 5% non-fat dry milk at 25°C for 30 min. Primary antibodies were diluted in 0.1% TBST and 3% bovine serum albumin (BSA). The primary antibodies included *anti*-pJAK2 (#3776, Cell Signaling Technology, USA), *anti*-JAK2 (#3230, Cell Signaling Technology, USA), *anti*-pSTAT5 (#9351, Cell Signaling Technology, USA), anti-STAT5 (#, Cell Signaling Technology, USA), *anti*-pAKT (#9271, Cell Signaling Technology, USA), *anti*-AKT (#9272, Cell Signaling Technology, USA), anti-pERK (#9101, Cell Signaling Technology, USA) *anti*-ERK (#9102, Cell Signaling Technology, USA), *anti*-EPOR (#sc-365662, Santa Cruz Biotechnology, USA), *anti*-β-Actin (Santa Cruz Biotechnology, USA), *anti*-Bax (Santa Cruz Biotechnology, USA), anti-cleaved Casp3 (Cell Signaling Technology, USA), and anti-cleaved Parp1 (Cell Signaling Technology, USA).

### 2.9 Hypoxia and re-oxygenation

The HT-22 cells were seeded onto 96-well or 6-well plates. The next day, HT-22 cells were placed inside a sealed air-tight container consisting of an anaeropack (Mitsubishi Gas Company, Tokyo, Japan), which can induce a hypoxic atmosphere by absorbing oxygen and generating carbon dioxide. The cells were maintained in the hypoxic condition at 37°C for 18 h and re-oxygenated for 3 h at 37°C and 5% CO_2_. Subsequently, the cells were treated with reEPO or the peptides for 19 h before viability analysis.

### 2.10 Terminal deoxynucleotidyl transferase (TdT) dUTP nick-end labeling (TUNEL) assay

We fixed the cells with 4% paraformaldehyde in PBS on a slide at 25°C, washed them with PBS, and then treated them with 3% H_2_O_2_ in methanol for 10 min. After washing with PBS, we treated the cells with 0.1% sodium citrate in 0.1% Triton X-100 on sample slides for 2 min on ice and washed again with PBS. Subsequently, we treated the cells with the TUNEL reaction mixture for 60 min at 37°C in the dark. After washing thrice with PBS, 50 μl of TUNEL peroxidase (POD) was added to the slides for 30 min at 37°C in the dark. After washing thrice with PBS, we added 100 μl of 3,3′-diaminobenzidine (DAB) substrate onto the slides for 10 min at 25°C. Finally, we washed the cells thrice with PBS and observed the TUNEL-stained cells using an optical microscope.

### 2.11 Middle cerebral artery occlusion (MCAO) and reperfusion

The protocol of this experiment was approved by the Animal Care and Use Committee of the Keimyung University School of Medicine. After 1 week of acclimatization, mice were anesthetized with 3% isoflurane in a mixture of 70% N_2_O and 30% O_2_, and the anesthesia was maintained with 1.5–2.0% isoflurane. After the neck skin incision, the bifurcation of the common carotid artery was exposed. The external carotid artery was ligated, and the internal carotid artery was carefully isolated from the vagus nerve. A microvascular clip was placed across the right common carotid artery. Through the external carotid stump, a 7–0 surgical nylon monofilament with silicon coat was advanced into the right internal carotid artery up to the origin of the middle cerebral artery. Laser Doppler flowmetry (Perimed 5000 system, Järfälla, Sweden) was used to confirm adequate induction of focal cerebral ischemia. During the surgery, rectal temperature was monitored and maintained at 37 ± 0.5 °C with a feedback-controlled heating pad (CMA 150, Stockholm, Sweden). The filament was left undisturbed for 90 min. Then, reperfusion was performed by withdrawing the monofilament suture with an intravenous injection of reEPO (2000 IU/kg) or ML1-h3 (0.3 mg/kg) solution in 0.3 ml saline. The restoration of the blood flow was confirmed by laser Doppler flowmetry. Animals were then placed in a warm box at 30°C for 3 h to avoid bias in the results due to hypothermia. The mice were sacrificed at the end of the reperfusion period for the subsequent experiments (24 h after reperfusion following MCAO).

### 2.12 Evaluation of brain injury

The brain was rapidly dissected out and coronally sliced into 2-mm-thick sections. Subsequently, they were stained with 2%, 2,3,5-triphenyl tetrazolium chloride (TTC) in saline for 20 min at 37°C. The infarct volumes of the TTC-stained brain sections were imaged with a digital camera and analyzed. Using NIH ImageJ software (NIH, USA), the intensity of TTC in MCAO- and no-MCAO-performed hemispheres were digitally measured, and the ratio (MCAO versus non-MCAO) was presented as a percentage.

### 2.13 Measurement of the hematopoietic effect

The protocol of this experiment was approved by the Animal Care and Use Committee of the DGIST (DGIST-IACUC-21090804-0000). After two weeks for quarantine and acclimatization, male mice (C57BL/6 N, Koatech, Korea) were subcutaneously injected with vehicle (n = 6), reEPO (n = 6, 150 IU/kg), or ML1-h3 (n = 6, 0.2 mg/kg) three times a week for four weeks. After three days of final injection, they were anesthetized by injection of zoletil (50 mg/kg), and their blood was gathered from posterior vena cava into an EDTA-coated tube (#367863, BD Vacutainer, USA) using a syringe. The blood samples were analyzed by an automatic hematology analyzer (#ADIVIA 2120i, SIEMENS Healthineers, Germany) in DGMIF (Korea).

## 3 Results

### 3.1 Short peptides derived from helix C of EPO have a neuroprotective effect

**Fig. 1:**
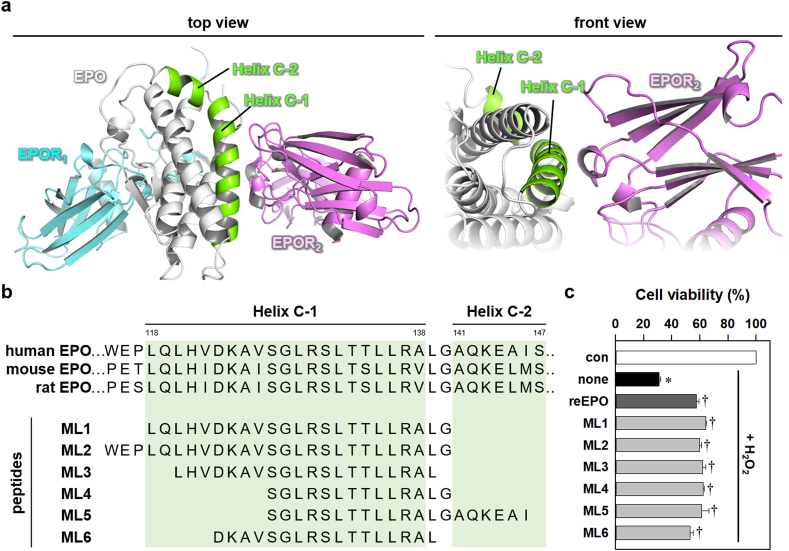
Neuroprotective effect of peptides spanning the helix C of EPO. (**a**) Front-view (left panel) of the three-dimensional structure (PDB #1EER) of processed human EPO (white cartoon) and extracellular chains of dimeric human EPORs (cyan cartoon, EPOR_1_; violet cartoon, EPOR_2_). An enlarged front-view (right panel) of low-affinity interface between helix C and EPOR_2_. Green helices indicate the region of helices C-1 and C-2. (**b**) The amino acid sequence of peptides spanning helices C-1 and C-2 of EPO. Amino acids are labeled by the one-letter symbol. (**c**) Cell viability of differentiated neuronal PC12 cells treated with 300 μM H_2_O_2_ for 24 h with or without reEPO (0.5 IU/ml) or the peptides (1 ng/ml). Groups labeled as con and none indicate control and no compound. (n = 3, *p<0.05 for con and †p<0.05 for none by *t*-test).

Previous studies have suggested that neuroprotective peptides, including Epotris, MK-X, and NL100, are derived from helix C of EPO upon low-affinity interactions with EPOR [21,35,42]. The helix C of EPOR-bound EPO curves into a very short loop (^139^Leu-^140^Gly) and is separated into helix C-1 (^118^Leu-^138^Ala/Val) and mini helix C-2 (^141^Ala-^147^Ser) at a low-affinity interface (Protein Data Bank #1EER, Fig. 1a and b) [35]. We synthesized several monomeric peptides spanning the helices C-1 and -2 (Fig. 1b), ML1 (^118^Leu-^140^Gly), ML2 (^115^Trp-^140^Gly), ML3 (^120^Leu-^139^Leu), ML4 (^127^Ser-^140^Gly), ML5 (^127^Ser-^146^Ile), and ML6 (^123^Asp-^139^Leu) regions. We then examined their cell-protective effects against H_2_O_2_-induced oxidative stress in differentiated neuronal PC12 cells expressing endogenous EPOR [43]. Consistent with the previous studies [21,35,42], we found that all the peptides exhibited neuroprotective effects similar to the reEPO (Fig. 1c). Even the shortest peptide without helix C-2 coverage, ML4, showed a neuroprotective effect. These results suggested that helix C-1 was the minimal and a critical region for neuroprotection. Based on these results, we selected ML1, which had the complete sequence of helix C-1 and a better neuroprotective effect than reEPO, as a prototype for second-generation EPO-derived peptide.

### 3.2 Divergent modification of EPO-derived peptides having differential affinity to EPOR

**Fig. 2:**
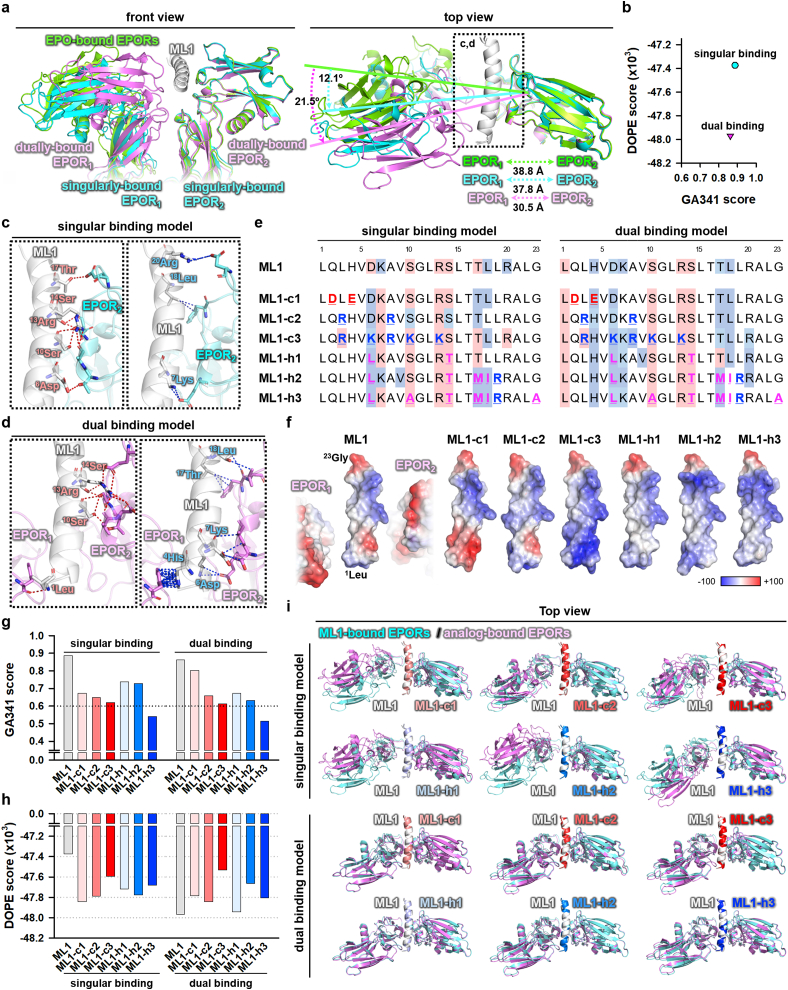
Design of ML1 and its analogs based on computational prediction. (**a**) Merged cartoon of potential singular and dual binding models of ML1 modified from PDB #1EER (white, ML1; green, EPO-bound EPORs; cyan and violet, singularly- and dually-bound EPORs with ML1). Change in the angle of EPORs visualized by curved spotted arrow (cyan or violet, singularly- or dually-bound EPORs with ML1 versus EPO-bound EPORs). Distance of EPORs is indicated by spotted bidirectional arrow (green, EPO-bound EPORs; cyan and violet, singularly- and dually-bound EPORs with ML1-h3). (**b**) DOPE and G341 scores in singular and dual binding models of ML1 and EPORs. (**c**, **d**) Enlarged images in the inset of (**a**) of potential polar (**c**) and proximal contacts within 4.0 Å (**d**) (crimson and sky, amino acids involved in potential polar and proximal interactions). (**e**) Amino acid sequences of ML1 and analogs (crimson- and sky-colored shades, amino acids involved in potential polar and proximal interactions). Bold, colored, and underlined letters indicate the modified amino acid (blue and red, positive- and negative-charged residues; magenta, hydrophobic residues). (**f**) Electrostatic potential on the molecular surface of ML1 and its analogs with EPOR_1_ and EPOR_2_ (red, negative-; white, neutral-; blue, positive-charge). For discrete visualization of electrostatic potential, EPOR1 and EPOR2 are separated from ML1. (**g, h**) Comparison of G341 (**g**) and DOPE (**h**) scores of potential binding models between ML1 and its analogs. (**i**) Merged images of potential binding models of ML1 and the peptides with EPORs (cyan and violet, ML1- and analog-bound EPORs).

We simulated the *in silico* binding between ML1 and EPORs by using the MODELLER program [34] and deduced the most potential singular binding model between ML1 and EPOR_2_, which showed a reliable genetic algorithm 341 (G341) score (Fig. 2a and b). Interestingly, an energy-minimizing algorithm of MODELLER produced an additional dual binding model between ML1, EPOR_1,_ and EPOR_2_; it exhibited a considerable decrease on discrete optimized protein energy (DOPE) score with a reliable G341 score, as compared to its cohort model of singular binding mode (Fig. 2a and b). Notably, this model showed a remarkable increase in the angle and proximity between dimeric EPORs compared to the singular binding model (Fig. 2a). In the singular binding model, polar contacts in ^6^Asp, ^10^Ser, ^13^Arg, ^14^Ser, and ^17^Thr of ML1 to EPOR_2_, and proximal contacts within 4.0 Å in ^7^Lys, ^18^Leu, and ^20^Arg (Fig. 2c), were consistent with the previous study [35]. Interestingly, in the dual binding model, *de novo* formation of polar and proximal contacts in ^1^Leu and ^4^His of ML1 to EPOR_1_ were predicted, respectively (Fig. 2d). Thus, we postulated that the affinity of the ML1 could be altered by charge or hydrophobicity modifications on the amino acids at the interface of EPORs. To examine this possibility, we generated divergent charge-modified (ML1-c1, -c2, -c3) and hydrophobicity-enhanced analogs (ML1-h1, -h2, -h3) from the ML1 prototype by replacing the amino acids without too many changes in the critical amino acids interacting with EPOR (Fig. 2e). Using *in silico* molecular modeling [44], we found that the binding site and properties of the peptides to EPOR vary according to the modifications (Fig. 2e), resulting from various changes in the electrostatic potential of the molecular surface in binding interfaces of the peptides with EPOR_1_ and EPOR_2_ as compared to ML1 (Fig. 2f). Like ML1, the peptides also showed an additional dual binding mode with a reliable GA314 score, except ML1-h3 (Fig. 2g). The GA341 score of ML1-h3 was below 0.6 in both models, which implied that its binding to EPOR could be very unstable or transient. ML1, ML1-h1, and ML1-h3 exhibited lower DOPE scores in the dual binding model, while ML1-c3 and ML-h2 showed lower DOPE scores in the singular binding model (Fig. 2h). Furthermore, ML1 and its analogs induced differential changes in the geometry of dimeric EPORs (Fig. 2i). Therefore, *in silico* modeling data showed that modification of ML1 could change, add, or remove some contacts to EPOR, and even alter the geometry of EPOR (Fig. 2e,h).

### 3.3 ML1 and its analogs have a differential affinity to EPOR

**Fig. 3:**
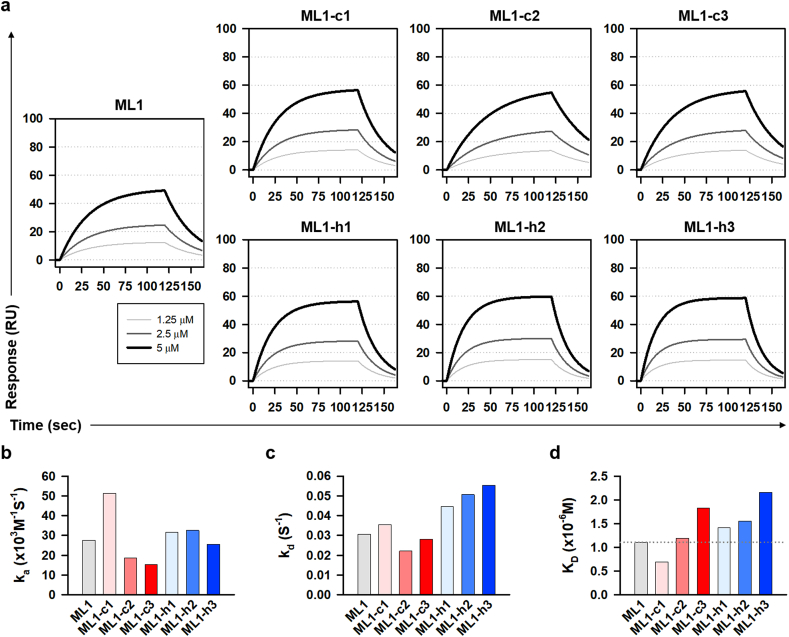
*In vitro* Binding affinity of ML1 and its analogs with EPOR. (**a**) Surface plasmon resonance spectroscopy (SPR) data for each peptide and EPOR. (**b-d**) Association rate constant (**b**), dissociation rate constant (**b**), and equilibrium dissociation constant (**d**) of peptides with EPOR, are measured by SPR.

We then investigated whether these modifications of ML1 practically affected the affinity to EPOR. From the surface plasmon resonance spectroscopy analysis, we found that all peptides could directly bind to recombinant human EPOR-Fc chimera protein in a dose-dependent manner (Fig. 3a). Interestingly, ML1 and its analogs exhibited different associations and dissociation kinetics with EPOR (Fig. 3b). ML1-c1 exhibited the highest association rate constant (k_a_), but ML1-c2 and ML1-c3 showed lower k_a_ than ML1 (Fig. 3c). ML1-h1, ML-h2, and ML-h3 exhibited higher dissociation rate constants (k_d_) than ML1 (Fig. 3d). These data indicate that perturbation of polar or proximal contact affects the association efficiency and stable complex formation between peptides and EPOR. Consequently, almost all ML1 analogs displayed a higher equilibrium dissociation constant (K_D_) than ML1, except ML1-c1 (Fig. 3e). This indicated a decreased affinity to EPOR. In particular, ML-h3 showed the weakest affinity to EPOR among the peptides, which was consistent with its GA341 score is below 0.6 *in silico* modeling (Fig. 2f). Therefore, these data suggested that ML1 and its analogs have unique *in vitro* agonistic potency to EPOR according to the properties of their interfaces with EPOR.

### 3.4 ML1 and its analogs have a differential effect on cell protection and proliferation

**Fig. 4:**
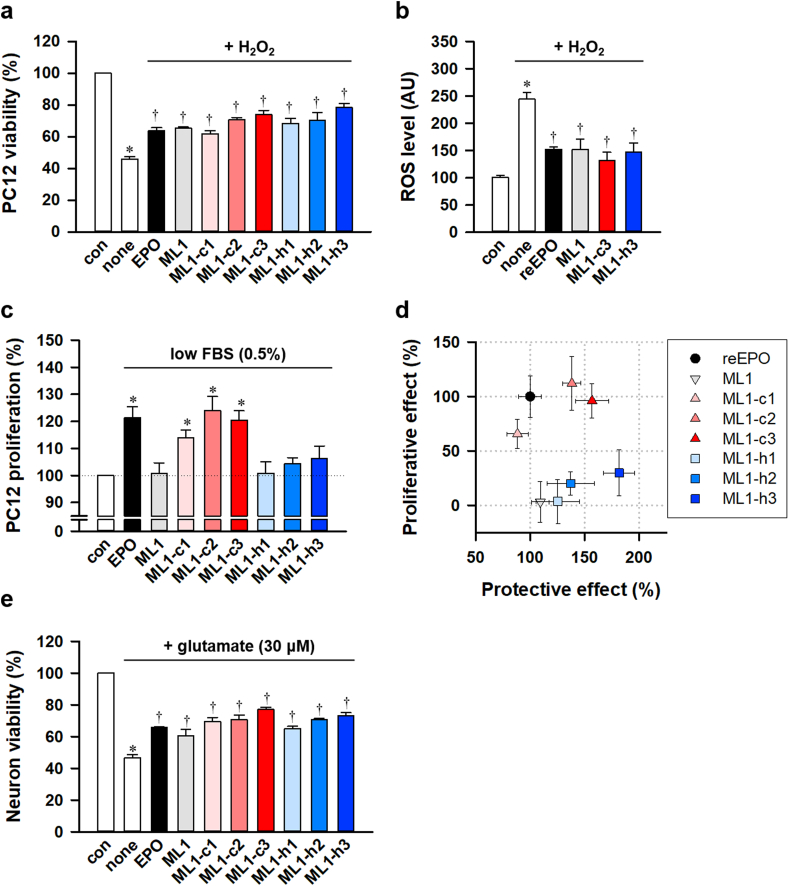
Differential effects of ML1 and its analogs on neuroprotection and cell-proliferation (**a**) Cell viability of differentiated neuronal PC12 cell upon H_2_O_2_ treatment for 24 h with or without reEPO (0.5 IU/ml), ML1 (1 ng/ml), or ML1 analogs (n = 5, *p<0.05 versus con and †p<0.05 versus none by *t*-test). (**b**) Level of ROS measured by H_2_DCFDA staining in differentiated neuronal PC12 cells upon H_2_O_2_ treatment for 6 h with or without reEPO, ML1, ML1-c3, or ML1-h3 (n = 5, *p<0.05 versus con and †p<0.05 versus none by *t*-test). (**c**) Cell numbers of undifferentiated PC12 cells with or without reEPO (10 IU/ml), ML1, or ML1 analogs (10 ng/ml) under low serum condition (0.5% fetal bovine serum) for 48 h (n = 5, p*<0.05 versus con by *t*-test). (**d**) Normalized protective and proliferative effects of reEPO, ML1, and its analogs (p*<0.05 versus reEPO by *t*-test). (**e**) Viability of cultured rat primary cortical neurons after glutamate treatment for 24 h with or without reEPO (0.5 IU/ml), ML1 (1 ng/ml), or ML1 analogs (1 ng/ml) (n = 3, *p < 0.05 versus con and †*p* < 0.05 versus none by *t*-test).

We examined the cell-protective effects of the new EPO-mimetic peptides. Like reEPO, treatment of all peptides significantly suppressed the cell death under H_2_O_2_-induced oxidative stress in PC12 cells (Fig. 4a). In addition, similar to reEPO, ML1, ML1-c3, and ML1-h3 decreased the levels of reactive oxygen species (ROS) induced by H_2_O_2_ (Fig. 4b). These data indicated that ML1 and its analogs have a cell-protective effect through ROS scavenging. Next, we assessed the cell-proliferative effect at the high concentration of the peptides under low serum conditions. We found that ML1 and hydrophobicity-enhanced analogs, ML1-h1, ML1-h2, and ML1-h3, did not induce proliferation in the PC12 cells. In contrast, charge-modified analogs, ML-c1, ML1-c2, and ML1-c3, significantly increased the proliferation, like reEPO (Fig. 4c). Remarkably, ML1-c3 and ML1-h3 had a stronger cell-protective effect than reEPO or ML1, by more than 150% and 180%, respectively (Fig. 4d). However, ML1-h3 exhibited no significant cell-proliferative effects, unlike reEPO and ML1-c3. These results indicated that the differential mode of associations and dissociations of peptides with EPOR, rather than their consequent value on affinity, affected the cell proliferation (Fig. 3). Furthermore, these peptides showed a neuroprotective effect against glutamate-induced excitotoxicity in cultured neurons like reEPO (Fig. 4e). As a result, we selected ML1-h3 as the most potential neuroprotective EPO-derived peptide without tumorigenic activity for further investigations.

### 3.5 ML1-h3 shows a differential mode of activation of EPOR signaling

According to our *in silico* prediction and several web-based prediction tools such as PEP-FOLD3 [45] and Jpred4 [46], ML1-h3 consists of amino acid sequences with an intrinsically high α-helical propensity on secondary structure. However, a previous study proposed that the helix C region of EPO, which is involved in low-affinity interaction with EPOR_2_, seems to have a little flexible or disordered propensity [47]. In addition, monomeric Epotris having a similar sequence to ML1-h3 showed disordered propensity [24]. According to *in silico* prediction of disorder score of IUPred3- and ANCHOR2 [48], N- and C-terminals of ML1-h3 have a potential disorder or flexible region. It can still be switched to the ordered structure by protein binding (Fig. S1a). Therefore, we examined whether ML1-h3 indeed has ordered α-helical structure by performing circular dichroism spectroscopy. Unexpectedly, the CD spectrum data predicted that ML1-h3 resolved in water solution showed less content of α-helix with a high content of other structures including β-sheet (Figs. S1b and c), similarly to monomeric Epotris [24]. Interestingly, upon gradual addition of trifluoroethanol, a negative-charged acidic solvent [49], its α-helix content was dramatically increased up to 65% with a reciprocal reduction of other contents (Figs. S1b and c). Considering that ML1-h3 can bind to recombinant human EPOR *in vitro* (Fig. 3), we suggest that α-helical folding of ML1-h3 require a suitable physiological conditions for protein-folding and receptor-binding [50].

**Fig. 5:**
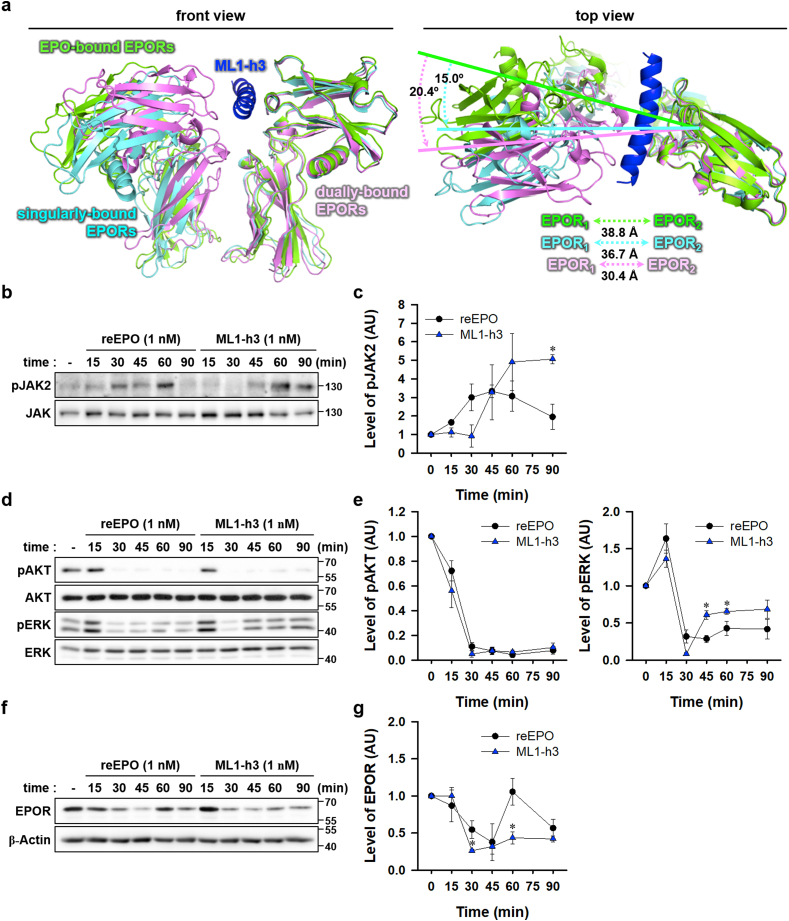
Differential activation of EPOR downstream signaling by reEPO and ML1-h3. (**a**) Potential models of EPOR geometry bound with ML1-h3 and EPO (blue, ML1-h3; green, EPO-bound EPORs; cyan and violet, singularly- and dually-bound EPORs with ML1-h3). Angles of EPOR in the upper panel are colored by cyan and violet, which indicate singularly- and dually-binding forms with ML1-h3. Distance of EPORs in the bottom panel is represented by green, cyan, and violet colors, which indicate EPO-bound form, singularly- and dually-bound forms with ML1-h3. (**b**) Time-series immunoblot of phosphorylated JAK2 (pJAK2) and JAK2 in undifferentiated PC12 cells upon treatment with 1 nM EPO and ML1-h3. (**c**) The relative level of pJAK2 level was measured and normalized by JAK2 level (n = 3, p*<0.05 for con by *t*-test). (**d**) Time-series immunoblots of phosphorylated AKT (pAKT) and ERK (pERK) upon treatment with EPO and ML1-h3 (**e**) Measurement of the relative level of pAKT and pERK, normalized by AKT and ERK levels, respectively (n = 3, p*<0.05 to con by *t*-test). (**f**) Time-series immunoblot of EPOR upon treatment with EPO and ML1-h3. (**g**) The relative level of EPOR, normalized by β-Actin level (n = 3, p*<0.05 for con by *t*-test).

A recent report has demonstrated that geometrical control of proximity and angle of dimeric EPOR by engineered ligands biases the activation of EPOR-downstream signaling pathways [38]. Through the computational modeling data, we found that ML1-h3 could increase the angle and proximity between EPORs (Fig. 5a). This result raised the possibility of a differential mode of action of ML1-h3 on EPOR signaling. We then examined how reEPO and ML1-h3 could activate JAK2 signaling at the same molecular concentration (1 nM), by immunoblotting. Treatment with reEPO upregulated the level of phosphorylated JAK2 in ^1007/1008^Tyr (pJAK2), an active form of JAK2 at 30–60 min; pJAK2 returned to the basal level at 90 min (Fig. 5a and b). ML1-h3 caused delayed elevation of pJAK2 level at 45 min, but it was maintained at 90 min (Fig. 5a and b). These data implied that reEPO and ML1-h3 exhibited differential kinetics for JAK2 activation. Next, we examined the downstream signaling pathway of JAK2. AKT and ERK signaling pathways are critical for EPO-mediated cell-protection and -proliferation through dimeric EPOR and/or the heterocomplex of EPOR and CD131 [51]. Like reEPO, ML1-h3 gradually reduced phosphorylated AKT in ^473^Ser (pAKT), which is active from AKT to a deficient level by 30 min, and the level was maintained at 90 min (Fig. 5c and d). reEPO and ML1-h3 acutely increased the level of phosphorylated ERK in ^202/185^Thr and ^204/187^Tyr (pERK), an active form of ERK, at 15 min (Fig. 5c and d). The level of pERK dramatically decreased to a level lower than the basal at 30 min and slowly recovered towards the basal level (Fig. 5c and d). Interestingly, pERK1/2 exhibited a more rapid recovery toward the basal level in ML1-h3 than reEPO treatment. These data indicated that ML1-h3 had differential activity for ERK1/2 but not AKT signaling. Previous studies have demonstrated that EPO-stimulated activation of EPOR is transient because EPO-bound EPOR undergoes rapid internalization and further degradation [52]. EPOR undergoes rapid turnover by recycling or trafficking internalized or *de novo* synthesized EPOR [53,54]. The level of EPOR protein was gradually decreased by reEPO for 15–45 min, and subsequently, it rapidly recovered to the basal level at 60 min (Fig. 5e and f). Interestingly, ML1-h3 induced delayed degradation of EPOR at 30 min and failed to restore the EPOR expression. Given that EPO-activated EPOR degradation depends on JAK2 activation [52], these data indicated that ML1-h3 induced suppression of EPOR recovery by long-lasting JAK2 activation.

### 3.6 ML1-h3 protects neurons in hypoxic conditions by suppressing oxidative stress *in vitro*

**Fig. 6:**
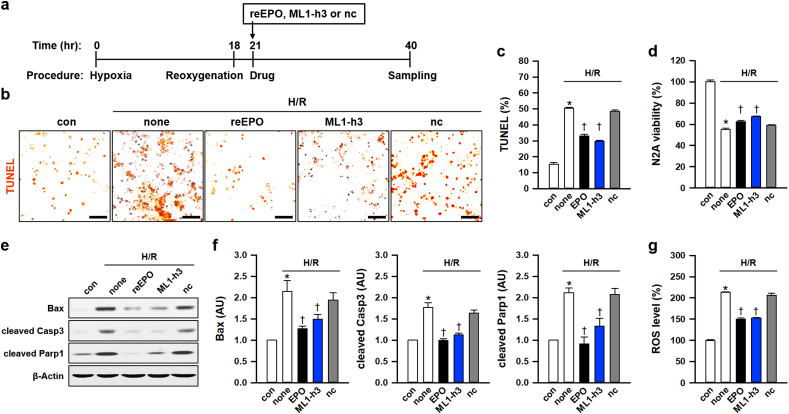
ML1-h3 has a cell-protective effect by mitigating ROS induced by hypoxia/re-oxygenation (**a**) Schematics depicting the procedure for hypoxia induction and reoxygenation with or without reEPO (0.5 IU/ml), ML1-h3 (1 ng/ml), or the negative control peptide (nc, 1 ng/ml) in HT-22 cells. (**b, c**) Representative images (**b**) for cell-population (**c**) of TUNEL-stained HT-22 cell after hypoxia and reoxygenation (H/R) with or without reEPO, ML1-h3, or nc (n = 5, *p<0.05 for con and †p<0.05 for none by *t*-test; scale bars, 50 μm). (**d**) Cell viability of HT-22 cell after H/R with or without reEPO, ML1-h3 or nc (n = 12, *p<0.05 for con and †p<0.05 for none by *t*-test). (**e**) Immunoblots of Bax, cleaved Caspase 3 (Casp3), and cleaved Parp1 in HT-22 cells after H/R with or without reEPO, ML1-h3, or nc. (**f**) Relative level of Bax, cleaved Casp3, or cleaved Parp1 normalized by β-Actin level (n = 6, *p < 0.05 for con and †*p* < 0.05 for none by *t*-test). (**g**) Level of ROS stained by H_2_DCFDA in HT-22 cells after H/R with or without reEPO, ML1-h3, or nc. (n = 12, *p<0.05 for con and †p<0.05 for none by *t*-test).

Next, we examined the neuroprotective effect of ML1-h3 under hypoxia and subsequent re-oxygenation (H/R) in mouse hippocampal cell-line, HT-22 (Fig. 6a). Similar to reEPO, ML1-h3 dramatically decreased the TUNEL-positive cells under H/R (Fig. 6b and c) and suppressed the loss of cell viability (Fig. 6d). These data indicated that ML1-h3 suppressed DNA damage and cell death under H/R. Previous studies have demonstrated that H/R evokes cell death by Bax and Parp1 activation, which mediate caspase-dependent and -independent cell death [55,56]. Immunoblot analysis showed that H/R induced upregulation of Bax and cleaved Caspase 3 and Parp1 (Fig. 6d). Notably, ML1-h3 efficiently reduced the levels of Bax, cleaved Caspase 3, and cleaved Parp1, similar to reEPO treatment (Fig. 6e). In addition, similar to reEPO, ML1-h3 also decreased ROS level induced by H/R (Fig. 6f), which indicated that ML1-h3 suppressed H/R-induced cell death by alleviation of ROS induction.

### 3.7 ML1-h3 mitigates brain injury in mouse ischemic model *in vivo*

**Fig. 7:**
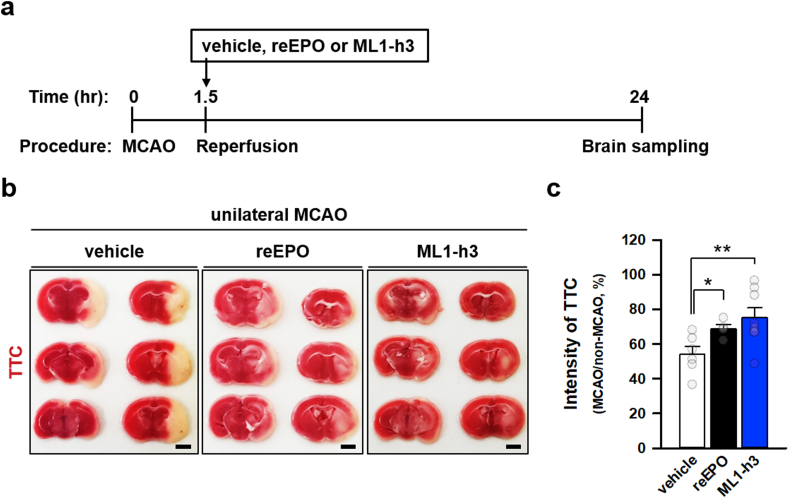
ML1-h3 suppresses brain injury in the *in vivo* mouse stroke model. (**a**) Schematic diagram of the procedure for MCAO in mouse and subsequent reperfusion with the injection of vehicle, reEPO, or ML1-h3. (**b**) Representative images of brain sections stained by triphenyl tetrazolium chloride (TTC) in mice performed by unilateral MCAO and reperfusion with the injection of vehicle, reEPO (2000 IU/kg), or ML1-h3 (0.3 mg/kg). MCAO is unilaterally performed for the right brain hemisphere, and the left hemisphere is on the contralateral side (scale bar: 1 mm). (**c**) Comparative levels of TTC in MCAO-performed vs contralateral side in vehicle- (n = 6), reEPO- (n = 4), or ML1-h3-injected mice (n = 8) (*p<0.05, **p<0.02 by *t*-test).

To further investigate the *in vivo* neuroprotective effect of ML1-h3 using mouse ischemic model, we performed unilateral middle cerebral artery occlusion (MCAO) and subsequent reperfusion in mice with the injection of EPO or ML1-h3 (Fig. 7a). Triphenyl tetrazolium chloride (TTC) staining showed that MCAO significantly decreased the average intensity of TTC by less than 54% in unilateral MCAO-performed side of the vehicle-injected mouse brain, as compared to the contralateral side (Fig. 7b). This indicated that MCAO induced extensive infarction in the brain. In contrast, injection of reEPO inhibited extinction of TTC-staining in MCAO-performed side (Fig. 7b and c) and showed a significant decrease in infarction by mitigation of brain injury. Strikingly, ML1-h3 strongly inhibited brain injury due to MCAO than reEPO, by more than 140%. Taken together, *in vitro* and *in vivo* experiments showed that ML1-h3 was a suitable neuroprotective peptide in neuronal injury models.

### 3.8 Role of ML1-h3 in the induction of hyper-erythropoiesis *in vivo*

**Fig. 8:**
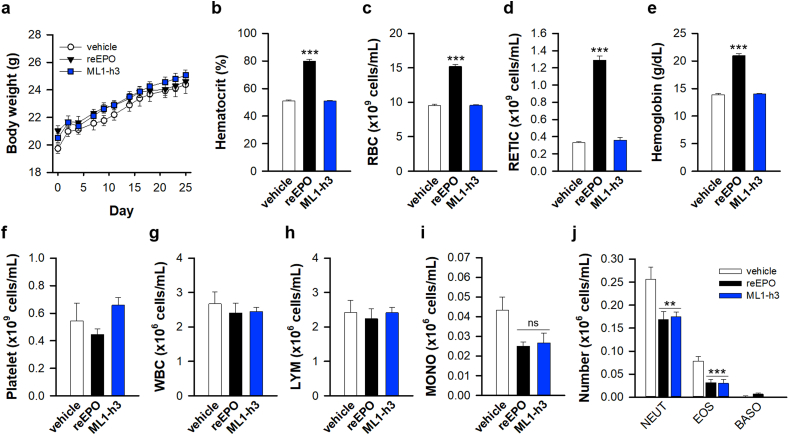
Effect of ML1-h3 on hematopoiesis. (**a**) Change of body weight of mice repetitively injected with vehicle, reEPO (150 IU/kg) or ML1-h3 (0.2 mg/kg) for 25 days. (**b-e**) Erythropoietic properties of blood in mice injected with vehicle (n = 6), reEPO (n = 6), or ML1-h3 (n = 6). Measurement of hematocrit (**b**), contents of red blood cell (RBC, **c**), reticulocyte (RETIC, **d**), and hemoglobin (**e**). (***p < 0.01 for vehicle by *t*-test). (**f-j**) Measurement of cell number of platelet (**f**), white blood cell (WBC, **g**), lymphocyte (LYM, **h**), monocyte (MONO, i), and granulocytes (neutrophil, NEUT; EOS, eosinophil; BASO, basophil) (***p < 0.01, **p < 0.02, ^ns^p>0.05 for vehicle by *t*-test).

Finally, we examined whether ML1-h3 is responsible for induction of hyper-erythropoietic effect *in vivo*. We repetitively injected reEPO or ML1-h3 into mice via subcutaneous route and analyzed for their hematological property. No significant difference was observed in body weight upon injection of reEPO or ML1-h3 (Fig. 8a). As anticipated, reEPO dramatically increased the hematocrit level, number of erythrocytes and reticulocytes, and content of haemoglobin, while ML1-h3 did not induce any significant change (Fig. 8b–e). However, both reEPO and ML1-h3 did not significantly affect the cell number of platelet (Fig. 8f), which is differentiated from megakaryocyte-erythrocyte progenitor with erythrocyte [57]. These data indicate that ML1-h3 does not have erythropoietic and megakaryopoietic effects, while reEPO intensively accelerates erythropoiesis. Recently, some immune cells are also known to express EPOR protein and reEPO that can regulate the proliferation and activity of immune cells [58]. Therefore, we also examined the effect on the population of immune cells. Injection of reEPO or ML1-h3 did not significantly affect the total number of white blood cells, lymphocytes, and monocytes (Fig. 8g–i). However, reEPO and ML1-h3 considerably decreased the number of granulocytes, including neutrophils and eosinophils (Fig. 8j), which was consistent with reports of a previous study [59]. These data indicate that reEPO and ML1-h3 induce moderate change in the generation of immune cells in our experimental model. We, therefore, suggest that ML1-h3 is a better potential neuroprotective compound than hyper-erythropoietic reEPO.

## 4 Discussion

The development of EPO-mimic peptide for substitution of EPO provides considerable benefits for biosafety, brain penetration, manufacture, and cost compared to reEPO and its variants, including AsialoEPO, EPOL, and carbamylated EPO. Previous ‘first-generation’ EPO-derived neuroprotective peptides including Epotris (^119^Gln-^138^Ala), NL100 (^127^Ser-^138^Ala), MK-X (^126^Val-^146^Ile), Epobis (^63^Asn-^80^Arg), and JM-4 (^55^Gly-^73^Val) were designed from original amino acid sequences of EPO. They showed sufficient neuroprotection without adverse outcomes such as erythropoietic effects [[20], [21], [22],^29^,^42^,^60^]. Epobis and JM-4 spanned the loop region between helices A and B, which are involved in high-affinity interaction with EPOR_1_. In contrast, Epotris, NL100, and MK-X are derived from helix C interacting with EPOR_2_. These indicate that partial binding to EPOR by either low- or high-affinity binding sites is sufficient for neuroprotective effect without adverse outcomes, including tumorigenic or erythropoietic effects [10,21,29,42,60]. Furthermore, these short peptides can efficiently cross the blood-brain barrier [21,29,42]. Thus, we aimed to develop a more advanced ‘second-generation’ neuroprotective peptide with an enhanced neuroprotective effect.

We selected ML1 from helix C of EPO as the prototype for the second-generation neuroprotective peptides. ML1 had exceptional structural properties as compared to the other similar peptides, including Epotris and NL100. Interestingly, *in silico* prediction data using an energy-minimization algorithm showed that ML1 and its analogs, through ^1^Leu could dually bind to both EPOR_1_ and EPOR_2_. Furthermore, this dual binding of the peptides to EPOR could induce intensive geometrical changes in dimeric EPORs compared to reEPO. However, other previously reported peptides derived from helix C of EPO, such as Epotris and NL100, do not contain the ^1^Leu. In addition, ML1 and its analogs were produced as monomeric form, while other previously reported peptides, such as Epotris and NL100, were produced as tetramer and dimer, respectively, using an additional linker [21,42]. Also, the peptides showed lower molecular weight (mw = 2440–2616 Da) than Epotris (mw = 9114.9 Da) and NL100 (mw = 2843.4 Da). Therefore, these molecular properties of ML1 provide more benefits in the aspect of manufacturing and cost.

Next, we generated various divergent analogs from ML1. Although further structural biological experiments are necessary for validating their binding to EPOR, *in silico* prediction and *in vitro* binding assay showed that ML1 and its analogs have a differential affinity to EPOR, thereby inducing a change in geometry of dimeric EPORs. In particular, ML1-c3 and ML1-h3 showed significantly reduced affinity to EPOR as compared to ML1. However, their association and dissociation modes were completely different. ML1-c3 containing positively charged amino acids on the interface with EPOR, showed a slow association rate with EPOR without any significant change in dissociation rate. In contrast, ML1-h3 added by hydrophobic amino acids exhibited unstable binding and rapid dissociation with EPOR without any considerable delay in the association. Interestingly, ML1-c3 and ML1-h3 had greater neuroprotective effects as compared to reEPO and ML1, respectively. However, unlike ML1-c3 and reEPO, only ML1-h3 exhibited no cell-proliferative outcome. Consequentially, we successfully generated divergent peptide analogs having differential effects via *in silico* and *in vitro* strategies. Regardless of these pharmacological and clinical potentials, some caution was required to interpret the biological data of geometric shift between the peptides and EPOR. First, since our *in silico* geometrical prediction and *in vitro* SPR analysis were based on only extracellular domains of EPORs, it is unclear how peptide binding induces the geometric event of membrane-spanned full-length EPOR. Second, endogenous EPOR, which would respond to our peptides, could be extracellularly glycosylated [[61], [62], [63]]. Although this glycosylation has been known to enhance the extracellular trafficking of EPOR rather than the binding affinity of EPO, its effect on binding with our peptides still remains in question. On the contrary, our CD spectrum exhibited that like monomeric Eportis, ML1-h3 forms disordered structures in water solutions [24], similar to Helix C region of EPO intrinsically that showed flexible or even disordered propensity, as proposed previously [47]. Notably, ML1-h3 was transformed into α-helix by adding TFE, implying that its α-helical folding can be seen in conditions suitable for protein-folding. We hypothesized that this disorder-to-order folding transition might require some molecular mechanisms [64]. EPOR-non-bound EPO forms a globular structure with well-ordered four α-helices (PDB #1BUY) [65]. Similarly, tetramerization of Epotris induces disorder-to-order transition into the α-helical structure, while monomeric Epotris showed disorder propensity [24]. These studies imply that the Helix C region has some flexibility and disorder propensity, and it can be transformed into a helix by molecular packing/crowding. Additionally, considering that ML1-h3 can bind to recombinant EPOR *in vitro,* we anticipate that ML1-h3 may undergo binding-driven folding to a-helix [66], even though the association is slow compared to reEPO (Fig. 3).

Although several EPOR-stimulating derivatives of EPO without erythropoietic or proliferative effects have been proposed over 20 years ago, the underlying mechanism has not been elucidated. The non-erythropoietic EPO-variants with absent or decreased sialic acids and EPO-derived peptides are rapidly cleared *in vivo* [[10], [11], [12]]. Conversely, erythropoiesis-stimulating EPO-variants exhibit a longer half-life in plasma [67]. These reports demonstrate that the erythropoietic effect is implicated in the duration of EPOR-stimulation. According to recent reports, engineered surrogate ligands, including diabodies and designed ankyrin repeat proteins, finely control orientation, proximity, or angle between dimeric EPORs. This process may induce a bias in activation of downstream signaling, different from reEPO, resulting in a differential cell-proliferative effect or selective erythropoiesis [38,68]. These reports suggest that EPO-derivatives may induce differential activation of EPOR by altering the geometry of dimeric EPORs. In this study, we found that the generated peptides, especially ML1-h3, could induce intensive geometrical changes in EPORs by dual binding mode *in silico*. ML1-h3 also exhibited delayed but prolonged activation of JAK2 as compared to reEPO, which resulted in suppression of EPOR recovery. Considering previous studies, this result may be due to alteration of the geometry of dimeric EPOR by ML1-h3 differently to reEPO [38,68]. In addition, we observed that ML1-h3 exhibited recovery in ERK activity to a higher level after peak activation than reEPO. Previous studies have shown that the activation period of ERK is critical for determining proliferation and differentiation of PC12 cells by epidermal growth factor (EGF) and nerve growth factor (NGF), respectively [69,70]. Recent live-imaging for ERK activation in single-cells showed that NGF induces heterogeneous ERK activation in each cell and a higher population of cells showing sustained ERK activation than EGF [71]. Based on these reports, we speculated that ML1-h3 induced more prolonged activation of JAK2 and ERK, unlike reEPO, and this process does not lead to cell proliferation.

Previous studies have demonstrated that CD131 functionally linked with EPOR mediates tissue protection by forming reEPO and EPO-derivatives [14,25,26,28,72,73]. In contrast, the cardioprotective effect of darbepoetin, a long-lasting EPO analog with low affinity to EPOR, is not inhibited in CD131-deficient mouse [74], indicating the tissue-protective effect of homodimeric EPOR. We assume that binding of ML1-h3 to CD131 may not occur directly as Epotris, derived from helix C of EPO, did not bind directly to CD131 *in vitro* [24]. However, *in silico* analyses of the EPO-bound complex of CD131-EPOR showed that helix C of EPO could associate with CD131 [12,75]. Therefore, although we postulated that the neuroprotective effect of ML1-h3 seems to be mediated by EPOR rather than CD131, we still could not exclude a potential involvement of CD131 by ML1-h3. More recently, Ephrin B4 receptor (EphB4) has been identified as an alternative receptor of EPO [76]. EPO enhances tumor growth and progression via activation of EphB4 signaling. This process limits the clinical usage of erythropoiesis-stimulating agents for cancer patients suffering from chemotherapy-induced anemia. Notably, helix C of EPO is involved in binding with EphB4 [76], implying that ML1 or ML1-h3 also may potentially bind to EphB4. Fortunately, considering that the binding affinity of EPO to EphB4 (K_D_ = 880 nM) is remarkably lower than to EPOR (K_D_ = 28 nM), we postulated that the affinity of peptides derived from helix C of EPO to EphB4 should be very low. Consistently with this postulation, we found that ML1-h3 has no cell-proliferative effect.

We also showed that ML1-h3 protects neurons through anti-oxidant effect observed *in vitro* hypoxia/re-oxygenation and *in vivo* ischemia/reperfusion models like reEPO. A mild hypoxic condition can evoke the neuroprotective response as preconditioning against brain injury, especially in ischemic stress [77]. This process requires hypoxia-inducible factor (HIF) signaling upregulating pro-survival proteins, such as EPO and BCL2 [[78], [79], [80]]. However, after severe hypoxia or ischemia, re-oxygenation or reperfusion induces intracellular calcium overload, ROS overproduction, and mitochondrial impairment, leading to activation of multiple cell death pathways, including apoptosis, autophagy, and necrosis [56,80]. In this context, HIF signaling paradoxically upregulates cell death-promoting proteins, including BNIP3 and p53 [81]. These studies show that a balance between pro-survival and pro-death signaling pathways mediated by HIF may determine cell fate. It has been demonstrated that administration of reEPO induces tissue-protective effect by inhibiting cell death pathway *in vivo* hypoxia/ischemia models of various tissues, especially the brain [82]. Similarly, we also showed that ML1-h3 inhibits Bax/Parp1-mediated cell death pathways like reEPO. On the other hand, re-oxygenation after preconditional hypoxia also evokes activation of the nuclear factor erythroid 2-related factor 2 (NRF2) signaling pathway, which is involved in cellular redox and defense system against oxidative stress and inflammation [[83], [84], [85]]. Upon mild oxidative stress during re-oxygenation, NRF2 stabilizes, translocates into the nucleus, binds onto antioxidant response element as a transcription factor, and finally activates transcription of anti-oxidant enzymes including heme oxygenase-1 with the expression of vitagenes such as heat shock proteins, thereby, protecting neurons [86,87]. Therefore, HIF and NRF2 signaling pathways may be involved in neuronal adaption against hypoxia/re-oxygenation and ischemia/reperfusion as endogenous ‘hormetic response.’ Hormesis is a biphasic response between pro-survival and cell death in a concentration/dose-dependent manner with cell/tissue-type-specific and temporal profiles [88]. Especially, a majority of anti-oxidant natural compounds and neuroprotectants, including polyphenolic phytochemical exogenously, evokes NRF2-mediated hormetic response, thereby, effectively reducing the occurrence and severity of various neurodegenerative diseases such as Parkinson's disease and Alzheimer's disease as well as ischemic stroke [88]. Similarly, administration of reEPO also can activate the NRF2 signaling pathway through ERK activation, resulting in neuroprotection *in vitro* neurotoxin models [89,90] and *in vivo* brain injury models including ischemia, trauma, and hemorrhage [[91], [92], [93], [94]], as well as an anti-aging effect [95,96]. These studies imply that NRF2 signaling pathways may mediate the anti-oxidant effect of ML1-h3, like reEPO. Furthermore, ML1-h3 can be used as a hormetic agent compared to EPO because of its non-erythropoietic and non-proliferative properties. As a result, ML1-h3 may enhance additional hormetic response for pro-survival and anti-oxidant signaling pathways, resulting in neuronal survival under pathological hypoxia/re-oxygenation and ischemia/reperfusion.

In conclusion, we suggested divergent modification approaches through computational simulation to develop novel ‘second-generation’ EPO-derived peptides for neuroprotection. However, as well as previously reported EPO-derived peptides, our peptides, including ML1-h3, still are in a native form, and this study leaves enough room for the development of more advanced EPO-derived peptides. So far, chemical modifications have been established to improve the stability and bioavailability of peptide drugs with advanced synthetic chemistry [97,98]. Acetylation at N-terminal, cyclization of N- to C-terminal, incorporation of N-methylated or unnatural amino acid, replacement to amide bond mimetics such as thioamides, peptoids, and β-amino acids, PEGylation, and lipidation have been used for peptide drug development. Therefore, we expect that these chemical modifications provide an important strategy for the development of ‘next-generation’ neuroprotective peptide drugs.

## Funding sources

This work was supported by the 10.13039/501100010274DGIST R&D Program of the Ministry of Science and ICT (21-BT-06), and the 10.13039/501100003725National Research Foundation of Korea (10.13039/501100003725NRF) Grant funded by the Korean Government (MSIP) (2014R1A5A2010008). It also was supported by the Basic Science Research Program of the 10.13039/501100003725NRF funded by the 10.13039/501100002701Ministry of Education (2020R1A6A1A03040516; 2021R1I1A3055783). The funding sources had no such involvement in study design, in the collection, analysis, and interpretation of data, in the writing of the report, and in the decision to submit the article for publication.

## Declarations of interest

None.
