# Supplementary material for: Second-generation non-hematopoietic erythropoietin-derived peptide for neuroprotection
Source: Redox Biol. 2021 Dec 21;49:102223. doi: 10.1016/j.redox.2021.102223 (PMC8715119; doi:10.1016/j.redox.2021.102223)
Supplement: Fig. S1 [file mmc1.pdf]

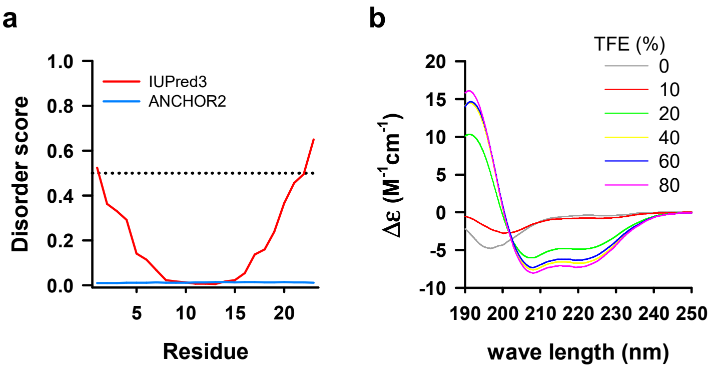

Supplementary figure 1. Analysis of secondary structure of ML1-h3 by circular dichroism spectroscopy (**a**) *in silico* prediction of disorder propensity on secondary structure of ML1-h3 based on IUPred3 and ANCHOR2 algorithms. (**b**) Circular dichroism spectra of ML1-h3 in water solution upon serial addition of trifluoroethanol (TFE). (**c**) BeStSel-based *in silco* analysis of content of a-helix, sheet, turn and others of ML1-h3 according to TFE concentration.
